# Interventional radiology in the management of benign biliary stenoses, biliary leaks and fistulas: a pictorial review

**DOI:** 10.1007/s13244-012-0200-1

**Published:** 2012-11-24

**Authors:** Miltiadis Krokidis, Gianluigi Orgera, Michele Rossi, Marco Matteoli, Adam Hatzidakis

**Affiliations:** 1 Department of Radiology, Cambridge University Hospitals NHS Trust, Hills Road, Box 218, Cambridge, CB2 0QQ UK; 2 Department of Radiology, S. Andrea Hospital Sapienza Rome University, Rome, Italy; 3 Medical School of Crete, Heraklion, Greece

**Keywords:** Biliary tract disease, Postoperative biliary injury, Benign biliary strictures, Bile leaks and fistula, Biliary drainage

## Abstract

**Background:**

Benign biliary postoperative stenoses and biliary leaks and fistulas usually occur due to injury after laparoscopic cholecystectomy, gastric or hepatic resection, bilio-enteric anastomoses and after liver transplantation. In most of the cases a new surgical intervention is not possible and the percutaneous trans-hepatic approach is of paramount importance in the diagnosis and treatment of the problem. This review aims to highlight the spectrum of percutaneous cholangiographic findings and methods of treatment of postoperative benign biliary stenoses and biliary leaks and fistulas. In the case of stenosis, dilation of the narrow tract is the usually the first approach, whereas in the case of leaks and fistulas bile diversion with drainage is usually attempted in order to seal the fistulous tract. However, a great variety of combination of materials and techniques may be used on a “case-by case” approach

**Methods:**

A selection of cases of benign biliary postoperative stenoses and biliary leaks and fistulas that were managed percutaneously are presented and the most common lines of approach are discussed.

**Conclusion:**

The imaging spectrum of percutaneous treatment of benign biliary postoperative stenoses and biliary leaks and fistulas is presented in order to aid interpretation and management with image guided procedures.

***Teaching Points*:**

• *Treatment of benign biliary stenosis is performed with cholangioplasty and stents*.

• *The main goal of fistula treatment is to divert the bile away from the site of bile wall defect*.

• *Drain collection and tract embolisation are the other options for bile leak percutaneous treatment*.

## Introduction

Interventional radiology (IR) has an established role in the diagnosis and management of patients with benign biliary disease. Postoperative benign biliary stenoses and/or biliary leaks and fistulas may be effectively treated with dilatation of the narrowed anastomotic tract or bile diversion away from the site of defect in the bile wall. A large variety of combination of dilatation and drainage may be used on a “case-by-case” basis with the use of various techniques and materials. Purpose of this pictorial review is to highlight the general lines of approach and to show the range of imaging findings of the percutaneous treatment of postoperative benign biliary stenoses and biliary leaks and fistulas.

## Etiology

Postoperative benign biliary strictures may occur as anastomotic strictures after surgical bile duct repair or liver transplantation, strictures secondary to intraoperative injury (most commonly during laparoscopic cholecystectomy) or postoperative inflammatory strictures [1]. Effective management is necessary in such cases in order to avoid serious consequences like liver function deterioration, cholangitis, jaundice, abscess formation and sepsis [1].

Endoscopic treatment is the optimal initial management for benign biliary strictures; however, the endoscopic approach is impossible for patients who have previously undergone bilio-digestive anastomosis [2] and may be very difficult in tight low biliary strictures [3]. In such cases, percutaneous treatment with balloon dilation with or without long-term biliary drainage and bare or covered stent placement, have been suggested as possible alternatives [2, 4–8].

Biliary leaks and fistulas are also a common complication after liver and biliary surgery. Leaks and fistulas may take origin from various procedures like bilio-digestive anastomoses, bile or cystic duct stumps or other intraoperative bile duct injury [9, 10]. Despite reduction in mortality for hepatic surgery in the last 2 decades, bile leaks rates have not changed significantly. Most bile leaks from the intra-hepatic biliary tree are transient and managed conservatively by drainage alone or by endoscopic biliary decompression. Minimally invasive percutaneous techniques for the management of biliary leaks and fistulas include percutaneous bile collection drainage, percutaneous trans-hepatic biliary drainage, biliary leak site embolisation sclerosis, ablation of a leaking biliary segment and treatment with a covered stent.

The most frequent causes of benign strictures of the biliary tree can be related to postoperative injury not only after open or video-laparoscopic cholecystectomy (VLC) but also after gastric or hepatic resection, portacaval shunt and bilio-enteric anastomosis [1]. Many factors, including inadequate exposure, congenital anomalies, obesity, hostile abdomen, acute pre-existing cholecystitis and bleeding, increase the rate of bile duct injury during cholecystectomy [11]. In addition, thermic injuries to the ductal wall produced by the propagation of heat during electrocutting or electrocoagulation may produce a progressive stenosis more often localised at the level of the bifurcation [12].

Liver transplantation (OLT) can represent a cause of injury to the biliary system including leaks and strictures. Strictures may be localised at the level of the anastomosis, in the intra-hepatic portion or diffuse, as seen in cases of hepatic artery thrombosis with an incidence of 10 % as reported by Zoepf et al. [13]. Moreover there is an increased incidence of subsequent diffuse biliary stenosis or “ischaemic cholangiopathy” in liver recipients. These patients can be treated either endoscopically or with radiological percutaneous approach with a success rate respectively of 100 % and 78 % [14]. The treatment selection is correlated to the initial surgical procedure and to the accessibility to the lesion.

Biliary leaks are an abnormal passage or communication from the biliary system to another location, intra- or extra-hepatic, and most commonly follow gallbladder surgery but can result from ductal injury related to blunt or sharp trauma or iatrogenic injury (e.g. liver biopsy) [15]. The most commonly accepted definition of a bile leak requires the presence of bile discharge from an abdominal wound and/or drain, with a total bilirubin level of >5 mg/ml or three-times the serum level, intra-abdominal collections of bile confirmed by percutaneous aspiration or cholangiographic evidence of dye leaking from the opacified bile ducts [16]. Surgery for hydatid disease may also lead to internal biliary leaks, with a frequency between 4 % and 28 %, mainly when deeply located cysts and right lobe cysts are excised [17].

The incidence of bile leaks following liver resection varies from 2 % to 30 % [18] and depends on the type, extent and reason for liver resection, with higher risk for intra-hepatic cholangiocarcinomas [19]. The risk of internal leak is higher after central hepatectomy involving segments IV, V and VIII, possibly due to drainage from aberrant right sided ducts draining into left duct.

## Clinical presentation

The clinical presentation of postoperative benign biliary stenoses may be divided into an early and late phase. The early phase is usually secondary to an acute obstruction of the bile duct and becomes clinically evident with an increase jaundice and alteration of liver function tests. Fever and infection may not be present, but may appear later.

The clinical presentation of biliary leaks and fistulas is usually with abdominal pain and raised inflammatory markers in the immediate postoperative period [20].

## Imaging methods and findings

Trans-abdominal ultrasound (US), computed tomography (CT) of the upper abdomen and magnetic resonance cholangio-pancreatography (MRCP) are the principal, non-invasive, imaging modalities in the evaluation of the liver parenchyma and the biliary tree.

MRCP is most frequently used in the case of a suspected stricture; the accuracy of MRCP versus the invasive endoscopic retrograde cholangio-pancreatography (ERCP) on the analysis of the type and site of obstruction is 76 % versus 72 % respectively [21].

In the case of bile leaks and fistulas, ultrasound and CT may help in the detection of the localised collections of bile or bile lying free in the peritoneal cavity. CT scanning may be used in conjunction with intravenous cholangiography (CT-IVC) to produce axial and three-dimensional images [22]. This technique is very useful in the detection of stones and in the creation of virtual cholangiographic pictures and may be very valuable in defining sites of leaks as it has the functional dimension that conventional MRCP does not.

However, if a leak is suspected, the best way to prove it is with the use of an invasive cholangiogram, either endoscopic or percutaneous. A cholangiogram is a dynamic exam that is able to demonstrate the origin of the leak and to delineate the fistulous tract [23] and is useful in deciding the optimal management and the likelihood of success with conservative measures. The most frequent site of leak demonstrated cholangiographically is the cystic duct stump post-laparoscopic cholecystectomy [24]. Radionuclide scans may also offer information in the case of bile leaks [25].

## Lesion classification

Biliary strictures and leaks are classified according to two widely used systems: the Bismuth classification, which is based on the location of stricture, and the Strasberg classification, which is based on the location of biliary leak [26, 27]. Bergman et al. [28] modified the Strasberg classification according to ERCP findings into four major types: Type A, leakage from cystic duct or peripheral hepatic radicles; Type B, leakage from a major bile duct; Type C, isolated ductal stricture; Type D, complete transection of the bile duct. Biliary leaks have been classified by Nagano et al. [16] into four types: Type A, minor leaks from small bile radicles on the surface of the liver; Type B, leaks from inadequate closure of the major bile duct branches on the liver’s surface; Type C, injury to the main duct commonly near the hilum; Type D, leakage due to a transected duct disconnected from the main duct.

## Percutaneous management of postoperative strictures

The main indications for a percutaneous treatment of a postoperative benign biliary stricture are: (1) poor patient’s general condition that does not permit another surgical intervention, (2) presence of altered anatomy that does not permit the endoscopic approach, (3) septic status that requires a quick approach for decompression of the biliary tree. The main contraindication is the presence of non-correctable bleeding disorder.

The treatment goals should be to improve and maintain bile duct patency as well as preventing stricture recurrence through a minimal invasive procedure. In the last 30 years percutaneous treatment of benign biliary strictures, with balloon dilatation and stenting, has been widely accepted as a valid therapeutic option with allow complication rate [29].

In the case of stenosis the biliary system is expected to be dilated and therefore easy to access percutaneously with US-guided puncture. However, in benign biliary stenosis often the ducts are not widely dilated as may be expected and US-guided puncture may not be straight forward. In such cases a central puncture with a 22-G needle be performed and obtain a cholangiographic picture and then perform a fluoroscopically guided puncture.

Once access in the biliary tree is obtained the next task will be that of crossing the stenosis with a guidewire. If a guidewire is advanced through the stenotic tract then there are two options: (1) advance an internal-external drain catheter and exchange the catheter gradually every 3 weeks to a larger diameter in order to dilate slowly the tract or (2) perform balloon dilatation of the stenotic area every 2-3 weeks upsizing the balloon in every session and having a 5-Fr access catheter left in situ. The presence of the internal-external drain is reducing the quality of life of the patient as this process may be long and require a period of 4–6 months and the external line is more prone to infections. In addition, the presence of a drain in situ helps quicker tract maturation.

In case of necessity of repeated dilatations for biliary stenoses and lack of endoscopic access instead of leaving an external access catheter a transjejunal approach may be performed. In such cases the procedure is performed via the fixed limb (either afferent or efferent limb) of a Roux-en-Y hepaticojejunostomy which had been created to repair biliary strictures or bile duct injuries, or specifically for the percutaneous biliary access to treat strictures or stones. The efferent limb of the Roux loop is surgically fixed to the peritoneum in a right anterior location, or the afferent limb is fixed anteriorly in the central upper abdomen. The loop can then be punctured under fluoroscopic or CT guidance usually with a thin needle; once access to the jejunum is obtained the biliary tree is catheterised in a retrograde fashion and bilioplasty may be performed. In the series by Fontein et al. [30] in 425 out of 494 (86 %) interventions the Roux loop was successfully accessed. The percentage of success was significantly higher for a fixed Roux loop (94 %) versus a non fixed loop (85 %). In only a very small percentage of cases a percutaneous trans-hepatic access was necessary. Interval cholangitis occurred in the 14 % of the patients and in eight cases postprocedural sepsis occurred.

Balloon dilatation is usually performed with an 8 or 10 mm wide and 2 to 4 cm long balloon. The size of the balloon is related to the type and location of the stenosis: a l0 mm balloon is generally used for common bile duct stenosis, while for lesions located in the intra-hepatic ducts 6 to 8 mm balloons may be selected. In case of biliary-enteric anastomoses, it is useful to perform the dilation using a larger balloon, varying from 10 to 20 mm in diameter; high pressure balloons may also be used and be inflated up to 22 Atm. Dilation needs to be slow and progressive in order to avoid laceration of the duct and massive bleeding into the biliary system and it may be repeated several times in particularly in case of fibrotic stenosis. The type of stricture is important in defining the response from dilalation. In the case of OLT anastomotic strictures (AS) appear to have a better response when compared with non-anastomotic strictures that are caused by ischaemic injury [31].

In recent years, the introduction of a cutting balloon (Boston Scientific, Natick, MA, USA) has been described in those patients with severe hard stenosis or fibrotic stenosis reducing the incidence of elastic recoil [32]. The cutting balloon is a non-compliant balloon with four blades at right angles to each other. When the balloon is inflated, the blades cut the adjacent fibrous tissue, while the non-compliant balloon stretches the duct wall increasing the inner luminal diameter. Certainly there is higher risk of complications like bleeding, perforation and also rupture of the biliary duct.

In case of biliary lithiasis—even though rare in a postoperative stenosis—dilation of the papilla must be performed to facilitate the passage of stones into the bowel by pushing through the papilla, into the bowel, stones usually less than 1 cm in diameter. In case of stones larger than 1 cm extracorporeal lithotripsy must be performed.

Recurrent stenosis after catheter or balloon dilation of benign biliary strictures may occur in 29–58 % of cases [29, 33]; to avoid this complication and to maximise the treatment outcomes, the treatment strategy may involve stent placement. However the role of stent placement after dilation is controversial because, while it may improve long-term success, the tube itself may stimulate inflammatory reaction, fibrosis and stone formation [32, 34]. Removal of the stent may be considered in the case of benign strictures that may be reached endocopically. The evaluation of the results before stent removal is usually based on cholangiographic-morphological criteria: decreased or normal calibre of the biliary ducts, disappearance of cholangitis and an absence of “filling defects” in the stenotic tract indicate a good result of the treatment. However, bare self-expandable metallic stents (SEMS) can create tissue hyperplasia and embedment, which would preclude stent removal, and so is not a recommended treatment for the benign condition. Therefore, covered metallic stents have been created and used in benign disease.

A study of 79 patients who underwent placement of partially covered metallic stents (Wallstent; Boston Scientific, Natick, MA, USA) for a variety of benign biliary stenoses including chronic pancreatitis, biliary stone disease, post-OLT and post-surgery showed that overall success rate was about 90 % after leaving the stents in place for 4 months. Stricture resolution rate was noted to be lowest in chronic pancreatitis. The authors were able to remove all stents after biliary decompression, as confirmed by clinical and laboratory investigation. Median follow-up time after stent removal was 12 months. Stent migration occurred in 14 % of cases, and tissue hyperplasia at the proximal uncovered portion of the stent, resulting in stricture was also noted in some patients [35]. To overcome the limitation of partially covered stents, fully covered self-expandable metal stents were developed and used in benign disease. The efficacy of covered stents in the treatment of anastomotic strictures following OLT is very promising. The stricture resolution rate ranges from 87.5 to 95.5 % at a follow-up period of 6–12 months. Recurrence of the stricture occurs in a very small number of patients. Stent migration is observed in many patients but no major complications have been reported [35, 36].

Covered stents have also the advantage that may be removed percutaneously; therefore, they are used in patients that are not suitable for endoscopic options [37]. In a retrospective comparison of percutaneously removed covered stents versus balloon dilatation in 66 patients, the primary patency of stents was 87 % at 3 years with a shorter catheter indwelling period. In two cases covered stent migration occurred and in one case the stent could not be removed [38].

## Percutaneous management of biliary leaks and fistulas

The goals of percutaneous treatment in biliary leaks and fistulas are: (1) to dilate any strictures, (2) to divert the bile away from the site of defect in the bile wall, (3) to drain thoroughly any collections and (4) to seal the tract if possible. Generally the option of treatment of biliary leaks follows the indication according to Nagano et al.’s [16] classification: Type A: these leaks are usually self-limiting, although sometimes ERCP and sphincterotomy may be required. Type B and C can be managed by ERCP and plastic stents combined with percutaneous drainage of the bile collection. Type D: require surgery and bilioenteric anastomosis or, if the draining segment is small, fibrin glue occlusion or acetic acid ablation. Sometimes operative excision of the excluded segment may be required.

**Fig. 1:**
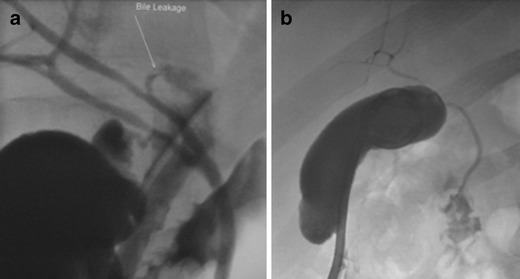
**a** Cholangiogram through a T-tube that shows the presence of a bile leak due to traumatic rupture of the main bile duct (*small arrow*). **b** The T-tube was exchanged to a locking pigtail drain catheter that drained externally. Cholangiographic control revealed that the leak was sealed a couple of weeks later

**Fig. 2:**
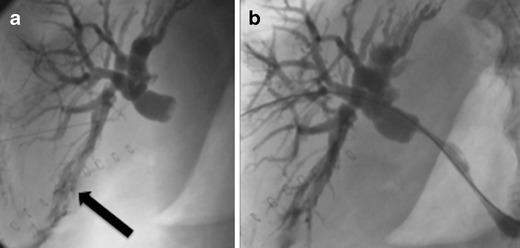
**a** Biliorrhea post open cholecystectomy occurred and ERCP failed to access the biliary tree due to concomitant stenosis of the common bile duct (CBD). Percutaneous puncture of the right side bile ducts and contrast injection. Cholangiogram revealed leakage from the ducts of Luscka (*black arrow*) and severe obstruction of the CBD. **b** The CBD was catheterised and a self-expandable metallic stent was deployed in order to drain the bile away from the side of leak

**Fig. 3:**
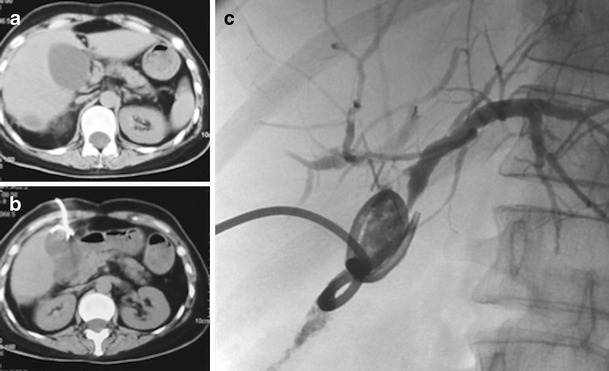
**a** Laceration and obstruction of common hepatic duct post laparoscopic cholecystectomy that led to biloma formation. **b** Percutaneous CT-guided drainage of the biloma. **c** Cholangiographic picture that confirms the communication of the collection with the biliary system. Surgical repair of the bile ducts followed

Usually a cholangiogram through the T-tube confirms the area of leakage. The T-tube may be exchanged to a large (12 or 14 Fr) locking pigtail, which may be left in situ for drainage until the fistulous tract is sealed (Fig. 1). In case of laceration of the ducts of Luscka ducts and concomitant bile duct obstruction, then dilation of the stenotic tract with a balloon or a stent might be necessary to heal the leak (Fig. 2). If laceration of large-calibre bile ducts occurred, then percutaneous drainage of the biloma for 2–3 weeks is suggested and eventual surgical repair of the leaking site is suggested (Fig. 3).

**Fig. 4:**
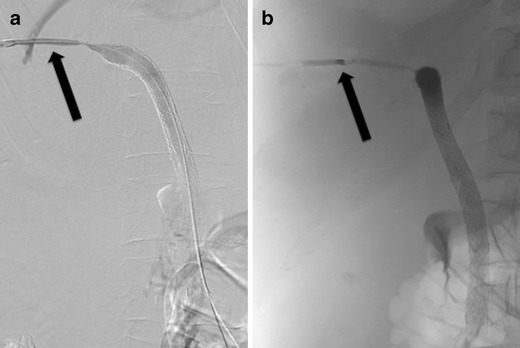
**a** Fistula between the bile ducts and a hepatic vein was revealed after a percutaneous biliary procedure (*black arrow*). **b** The fistulous tract was embolised with a pellet of Gelfoam (*black arrow*)

**Fig. 5:**
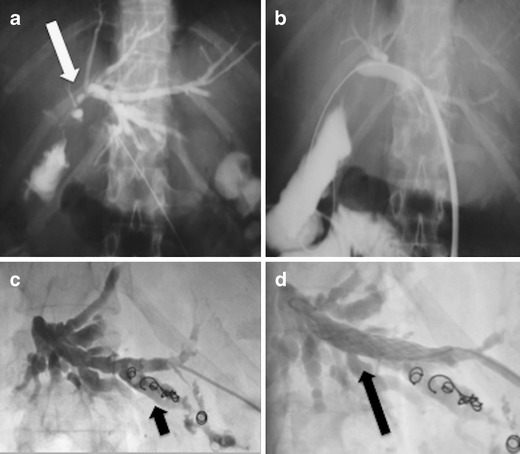
A patient with intra-hepatic stenosis and bile leak post biliodigestive anastomosis. **a** Percutaneous access that revealed the stenotic area (*arrow*). **b** Dilation of the stenotic tract. **c** The patient returned 9 years later with a new leak that was initially embolised with coils (*black arrow*) and then (**d**) a covered stent was deployed to seal the leak

In case of bilio-vascular fistulas embolisation with a variety of embolic agents may be performed (Fig. 4). If there is a fistula of the bile ducts with the hepatic artery, then biliary or arterial approach may be performed in order to embolise the fistulous tract. In such cases, patency of the portal vein needs to be checked, otherwise liver failure may be caused. The suggested embolic is a coil or micro-coil. In case of a biliary approach, antibiotics need to be administered due to the risk of abscess formation (Fig. 5).

In case of a free leak, a covered stent may also be used and then retrieved percutaneously. Gwon et al. [39] in a series of 11 patients with leaks after bilio-digestive anastomoses removed the covered stents after a mean time of 31 days and did not notice any recurrence in the 1 year follow-up.

**Fig. 6:**
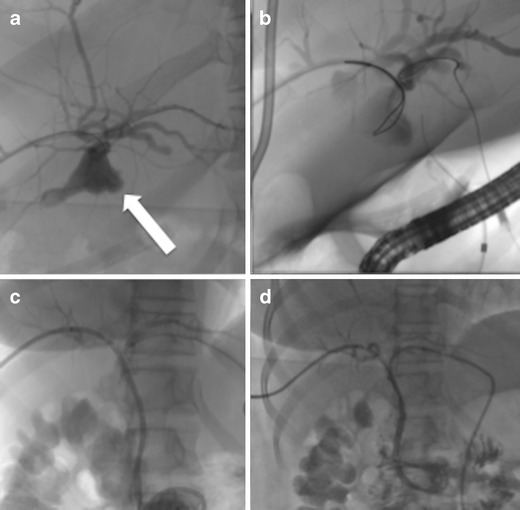
Complete avulsion of the bile ducts post cholecystectomy, with the presence of a bile leak outside the biliary tree. **a** Cholangiogram that reveals the area of bile leakage (*white arrow*). **b** A “rendezvous” procedure was performed with percutaneous and endoscopic access, and (**c**, **d**) restored the connection of the bile ducts with the duodenum

In case of “major” bile duct injuries, with complete transection, bile leakage, local fluid collection and inflammation surgical repair is extremely challenging, especially due to the compromise clinical conditions of the patient with fever, abdominal pain and bile fluid collection into the peritoneal cavity. In such cases, a radiological and endoscopic “rendezvous” may be performed in the first instance (Fig. 6). Fiocca et al. [40] described the results of this technique has been described in a study of 22 patients with complete transection of the common bile duct after cholecystectomy. This study showed that after a mean follow-up period of 4 years 16 patients were still asymptomatic and only four patients undergone surgery.

## Outcomes, complications and follow-up

Recurrence of benign stenosis after cholangioplasty may be due to chronic inflammation or possibly to poor stricture compliance owing to an elastic component in the scar tissue. In most series success is defined as the absence of symptoms following stent removal with normal bilirubin and alkaline phosphatase levels. Success rates ranging from 70 % to 100 % at a mean follow-up of 23–59 months (range 6–78 months) have been reported in the literature [13, 28, 31, 35]. However, a lower success rate (55 %) is reported in series with longer follow-up (5–7.5 years) [41, 42].

Most early procedure-related complications are associated with the trans-hepatic approach. Vascular complications have been reported in 2–20 % of patients undergoing percutaneous biliary puncture, in most cases resulting in transient haemobilia not requiring blood transfusion or intervention [43, 44]. Major complications are: septic shock (1.2 %) [43], septicaemia (5 %) [45] and haemorrhage requiring transfusion or hepatic artery embolisation (4 %) [36]. Episodes of cholangitis can occur while the stent is in place (1/17 cases) or after it has been removed (2/17 cases), and have been treated with intravenous antibiotics [29].
